# Systemic glucocorticoids use in post-COVID-syndrome patients does not affect retinal microcirculation

**DOI:** 10.1007/s10456-024-09926-8

**Published:** 2024-05-03

**Authors:** T. Kuchler, C. Schmaderer

**Affiliations:** School of Medicine, Klinikum Rechts der Isar, Department of Nephrology, Technical University of Munich, Ismaninger Str. 22, 81675 Munich, Germany

## Rational and baseline characteristics

We want to thank Peng et. al for the very thoughtful and important comments on our previous publication. They postulated that the utilization of systemic glucocorticoids might exert an influence on endothelial health in individuals previously afflicted with SARS-CoV-2 infection, particularly those treated in the acute phase with high doses of corticosteroids such as dexamethasone. Given existing literature suggesting the potential impact of high-dose corticosteroids on endothelial function, this hypothesis warrants further investigation.

In our cohort study, 4 patients (9.8%) were found to still be taking systemic corticosteroids at the time of analysis. 2 patients (4.8%) had received hospital treatment involving high-flow oxygen, indicating a potential indication for dexamethasone administration (with one patient fulfilling both criteria). Unfortunately, the precise administration of systemic glucocorticoids during the hospitalization phase among these patients was not documented. Nevertheless, given that the majority of our cohort experienced a mild acute infection, it is reasonable to estimate that only a minority received systemic glucocorticoid treatment during acute SARS-CoV-2 infection.

To address this knowledge gap, we conducted a comparative analysis between patients who are either currently using low doses of steroids or had received systemic glucocorticoid treatment and those with no history of glucocorticoid usage.

A total of five patients (mean age 47.0 years ± 7.9, 60.0% female) were identified as having received systemic glucocorticoid treatment (GC). When compared to glucocorticoid-naïve post-COVID syndrome (PCS) patients, those who had received GC showed a significantly more severe acute SARS-CoV-2 infection. Additionally, GC patients displayed higher C19-YRS scores, which is an established measure of PCS symptom severity. Although not reaching statistical significance, there was a trend towards elevated levels of markers associated with acute and chronic infection, such as ferritin and IL-6, in GC patients. Notably, there was also a tendency towards increased levels of vascular endothelial growth factor (VEGF) in GC patients (Table 1).

**Table 1:** Changes of retinal microcirculation in PCS patients with systemic glucocorticoids

Baseline characteristics			
Clincial characteristics	No syst. Steroid use (n = 36)	Syst. steroid use (n = 5)	*p* value
Age (years)			
Years, mean (SD)	41.5 (± 12.7)	47.0 (± 7.9)	0.36
Gender			
Female	28 (77.7%)	3 (60.0%)	0.58
BMI			
(kg/m^2^), mean (SD)	24.1 (± 3.8)	24.9 (± 5.1)	0.66
Obesity	3 (8.3%)	1 (20.0%)	0.42
Hypertension	6 (16.7%)	2 (40.0%)	0.25
Hypercholesterolemia			0.60
*Acute SARS-CoV-2 infection*			
Number of infections	1.3 (± 0.5)	1.2 (± 0.4)	1.0
Hospitalization acute infection	2 (5.6%)	2 (40.0%)	0.066
Severity acute infection			
2	22 (61.1%)	2 (40.0%)	0.004
3	12 (29.3%)	1 (20.0%)	
4	2 (5.6%)	0 (0.0%)	
5	0 (0.0%)	1 (20.0%)	
6	0 (0.0%)	1 (20.0%)	
Number of vaccinations			
0	3 (100.0%)	0 (0.0%)	0.46
2	12 (80.0%)	3 (20.0%)	
3	21 (91.3%)	2 (8.7%)	
	*PCS characteristics*		
Sick leave	26 (72.2%)	4 (80.0%)	1.00
PCS duration			
months, Mean (SD)	12.0 (± 7.9)	17.6 (± 11.6)	0.17
Job loss	8 (22.2%)	0 (0.0%)	0.56
Sick leave (days)			
days, Median (IQR)	119.0 (0.0–291.0)	195.5 (169.8–410.5)	0.16
	*Comorbidities*		
Depression	10 (27.8%)	1 (20.0%)	1.0
Asthma	5 (13.9%)	1 (20.0%)	0.57
Hypothyroidism	8 (22.2%)	0 (0.0%)	0.56
*Severity Scores* FatigeuSeverity.Scale			
Median (IQR)	6.0 (4.7–6.6)	6.6 (6.1–7.0)	0.12
C19-YRS			
Mean (SD)	36.03 (± 17.69)	53.40 (± 15.57)	0.044
PCS Severity			
Mean (SD)	35.72 (± 9.79)	35.50 (± 10.41)	0.96
PHQ9			
Mean (SD)	10.60 (± 4.64)	11.60 (± 3.21)	0.65
*Lab parameters*			
Ferritin			
ng/ml, Median (IQR)	95.0 (52.0–144.0)	174.0 (151.0–214.0)	0.082
Missing	4 (10.8%)	0 (0%)	
Leucocytes			
(G/l), Median (IQR)	6.2 (5.3–7.0)	8.3 (7.0–9.3)	0.16
Missing	4 (10.8%)	0 (0%)	
CRP			
(mg/dl), Median (IQR)	0.1 (0.1–0.1)	0.1 (0.1–0.4)	1.0
Missing	3 (8.1%)	0 (0%)	
IL-6			
pg/ml, Median (IQR)	19.9 (15.2–27.1)	30.0 (23.1–37.0)	0.20
CXCL10			
pg/ml, Median (IQR)	38.1 (31.0–45.9)	25.6 (24.3–37.9)	0.39
Missing	3 (8.1%)	0 (0%)	
IL-8			
fg/ml, Median (IQR)	1784.2 (1298.2–2714.1)	1529.8 (1071.1–1891.0)	0.48
Missing	3 (8.1%)	0 (0%)	
D Dimer			
μg/l,Median (IQR)	264.0 (200.0–426.8)	319.5 (277.5–396.2)	0.40
Missing	5 (13.5%)	0 (0%)	
VEGF			
Mean (SD)	7.1 (± 9.6)	16.7 (± 21.8)	0.089
Missing	1 (2.8%)	0 (0%)	
ICAM			
Mean (SD)	2398.1 (± 1268.1)	2691.4 (± 629.8)	0.62
Missing	1 (2.8%)	0 (0%)	

To partially answer the question, we compared retinal vessel analysis (RVA) parameters in PCS patients with GC use versus those without. We found no significant differences in venular (vFID) or arteriolar dilation (aFID) between the two cohorts.

**Fig. 1:**
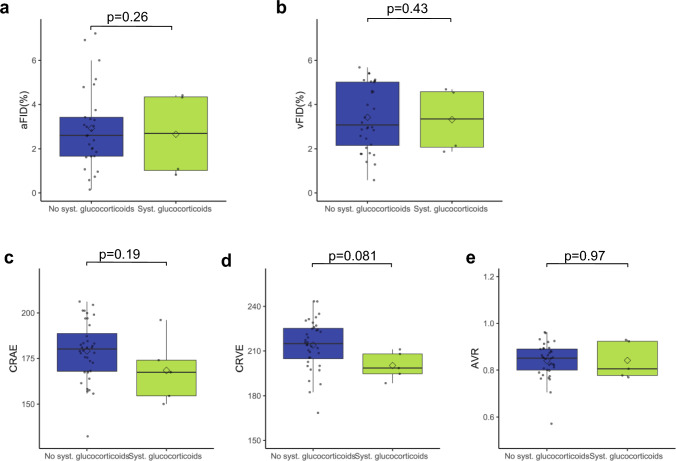
Parameters of retinal microvasculature in PCS patients with and without systemic CS use. Boxplots show arteriolar dilatation (aFID) and venular dilation (vFID) (**a** and** b**) and SVA parameters CRAE, CRVE and AVR (**c**–**e**) in patients without CS use (blue) and with CS use (green). Mean values are shown as a rectangle and median values as a line. To compare groups Wilcoxon rank sum test was used for skewed data and Welch´s t-test for normally distributed data

For static retinal analysis, the central retinal arteriolar equivalent (CRAE) tended to be lower in GC patients (179.1 ± 16.3 vs. 168.4 ± 18.2, p = 0.19), although this difference did not reach statistical significance. Similarly, the central retinal venular equivalent (CRVE) also showed a tendency to be lower in GC patients (213.9 ± 16.7 vs. 200.2 ± 9.3, p = 0.081), but again did not reach statistical significance. Given that both CRAE and CRVE were lower in GC patients, the arteriovenous ratio (AVR) was not significantly altered in patients using glucocorticoids (0.84 ± 0.07 vs. 0.84 ± 0.08, p = 0.97) (see Fig. 1).

As noted by Peng et al., potential confounders such as arterial hypertension, obesity, age, gender, and nicotine abuse may influence retinal microcirculation. After controlling for these confounders, our observations still did not show a significant association with systemic glucocorticoid use (see Table 2 a, b, and c).

.

**Table 2:** Association of SVA with CS use. SVA parameters as the dependent variable

Characteristics	Univariate		Multivariate^b^		R^2^/R^2^ adjusted Coefficient
	β-Coefficient	P-value	β-Coefficient	P-value	
Arteriolar venular ratio (AVR) as the dependent variable					
Age, year	− 0.0002	0.77			
Gender, male	− 0.02	0.44			
Obesity, year	− 0.07	0.082			
Art. hypertension	− 0.02	0.47			
Nicotine abuse	− 0.02	0.38			
Syst. Glucocorticoids	0.02	0.64	0.02	0.64	0.20/0.06
Central retinal arteriolar equivalent (CRAE) as the dependent variable					
Age, year	− 0.11	0.63			
Gender, male	− 5.1	0.16			
Obesity, year	− 11.4	0.20			
Art. hypertension	5.0	0.46			
Nicotine abuse	11.0	0.14			
Syst. Glucocorticoids	− 11.0	0.19	− 8.4	0.31	0.19/0.05
Central retinal venular equivalent (CRVE) as the dependent variable	− 0.05	0.34			
Age, year	5.8	0.83			
Gender, male	3.0	0.74			
Art. hypertension	7.6	0.25			
Nicotine abuse	16.9	0.02*			
Syst. Glucocorticoids	− 13.8	0.081	− 15.0	0.058	0.27/0.14

## Discussion

Systemic glucocorticoid use can adversely affect endothelial function through multiple mechanisms, including impaired NO signaling, increased oxidative stress, alterations in inflammatory and immune responses, changes in vascular permeability, prothrombotic effects, and vascular remodeling. In our cohort four patients still used systemic GC up to the recruitment timepoint and potentially two patients (with one patient overlapping) were treated with high dose GC during their acute infection. The evaluation in our cohort showed no significant alterations neither in static parameters nor in dynamic vessel analysis. As our cohort size is very small effects of GC on retinal microcirculation would need to be substantial for us to observe them. One could also argue that in states of inflammation GC treatment may even improve endothelial function. One study in giant cell arteritis patients could show significant improvement of endothelial function after application of GC shown by improvement of brachial artery flow-mediated dilatation [1]. As ongoing inflammation is one of the postulated hallmarks of PCS GC use could also be beneficial. Cervia-Hasler et al. recently suggested that complement dysregulation and thromboinflammation with subsequent endothelial dysfunction may play a significant role in the pathophysiology of PCS [2], making glucocorticoids a potential therapeutic option. Regarding the potential effects of GC in post-COVID syndrome there are also studies showing associations between GC and improvement of Long COVID symptoms particularly in patients with initial abnormalities on CT and resting hypoxia or exertional desaturation [3]. Especially patients with persistent pulmonary affection seem to benefit from oral GC [4]. In our small cohort, we were unable to detect significant differences in retinal microcirculation. Limitations include the very small number of treated patients. Given the lack of treatment options for PCS patients, further investigations into glucocorticoid use would be of great interest. However, due to the potential side effects of glucocorticoids and their dual role in endothelial health, careful selection of target patients and indications for treatment will be challenging.
